# The influence of screen time on children’s language development: A scoping review

**DOI:** 10.4102/sajcd.v69i1.825

**Published:** 2022-02-10

**Authors:** Nazeera F. Karani, Jenna Sher, Munyane Mophosho

**Affiliations:** 1 Department of Speech Pathology, Faculty of Humanities, University of the Witwatersrand, Johannesburg, South Africa

**Keywords:** language development, screen time, children, positive effects, negative effects

## Abstract

**Background:**

An exponential increase in screen time amongst children and adults, has given rise to a plethora of studies exploring the influences that this exposure may have on children’s development.

**Objectives:**

This review is specifically concerned with understanding the influence of screen time on children’s language development.

**Method:**

A scoping review was conducted to explore the available literature relating to the impact of screen time on children’s language development. The scoping review was based on the Preferred Reporting Items for Systematic Reviews and Meta-analyses extension for Scoping Reviews (PRISMA-ScR) framework and the Joanna Briggs Institute (JBI) framework. The data were analysed using thematic content analysis.

**Results:**

This review identified 12 articles. It made an argument for the multifactorial relationship between screen time and language development, given the associated positive and negative effects. The results revealed core themes such as the influence of screen time being dependent on various factors and the diverse effects of screen time on children’s language development, with the inclusion of parents’ monitoring of and participation in viewing, playing a vital role in language development.

**Conclusion:**

The review indicated that an increase in the amount of screen time and an early age of onset of viewing have negative effects on language development, with older age of onset of viewing showing some benefits. Video characteristics, content and co-viewing also influences language development. This study demonstrates that the negative influences of screen time appear to outweigh the positive influences.

## Background

The fourth industrial revolution (4IR) has led to an increased exposure to screen time in both children and adults globally (Kardefelt-Winther, 2017; Rideout & Hamel, 2006). South Africa has similarly shown an escalated usage of screen time amongst children and adults (Muthuri et al., 2014). The increased screen exposure may influence the overall development of children. According to Nurturing Care: For Early Childhood Development (2020), 38% of children were at risk of poor development in 2015. In addition, no data is available in South Africa relating to early stimulation, children’s books and playthings in the home environment (Nurturing care: For early childhood development, 2020). These are especially vital for children’s language development in the first 1000 days (3 years) of life, as this is a critical stage for brain development and maturation, and is the most intensive period of acquiring speech and language skills (Cusick & Georgieff, 2016). However, because of the increase in screen time in children, its influence on language development may be an area that needs to be explored.

According to current literature, ‘screen time’ can be defined as the duration of time that is spent with any screen such as phones, video games, televisions, computers, laptops and tablets (Ponti et al., 2017). In addition, ‘screen time’ may refer to either active or passive screen time (Sweetser, Johnson, Ozdowska, & Wyeth, 2012). Active screen time is the child’s ability to engage cognitively or physically in digital activities (Sweetser et al., 2012), whereas passive screen time includes inactive screen-based activities and/or obtaining digital (screen-based) information in a passive manner (Sweetser et al., 2012). It is important to distinguish between active and passive screen time as it will assist in understanding the specific effects of screen time (i.e. either positive or negative).

According to a review of research on physical activity, sedentary behaviour, nutrition, and overweight in children and adolescents (3–18 years old) from South Africa conducted by the Sports Science Institute of South Africa (2018), children in rural areas spend 70% of the day sedentary, which is comparable to children attending preschools in low- and high-income urban areas spending approximately 73% of the preschool day sedentary (Draper et al., 2019). Children in low- and high-income urban areas, as well as rural areas in South Africa exceeded the limit of two hours of screen time per day (with pre-schoolers having a limit of 1 h per day) and are exposed to over three hours daily, excluding that for schoolwork (Draper et al., 2019). According to the studies in the United States (US), this may be because of parents in the rural and urban areas allowing their children to watch television as it acts as a ‘babysitter’ while the parents are at work or occupied. Furthermore, screen time may be perceived to be educational and beneficial to their child’s brain development (Ruangdaraganon et al., 2009).

The review of the literature revealed both positive and negative effects of screen time on children’s development (Ruangdaraganon et al., 2009). The link between screen time and speech and language development is not straightforward, and several factors need to be considered. These factors include the duration of screen time, the presence of a co-viewer, video characteristics, and additional factors that may affect language. This will be elaborated on further under discussion.

The positive effects include educational value, expansion of vocabulary, exposing children to various experiences and cultural and linguistic diversity, and keeping them occupied in a safe manner (Balton, Uys, & Alant, 2019; Jordan, 2005; Rideout & Hamel, 2006). While these studies have reported positive effects, others have reported negative effects on speech, language, motor, cognitive and social development. Children begin understanding information after the age of two and may have trouble transferring information learnt from a device’s screen (Ponti et al., 2017). Therefore, it is important for children to be stimulated and that they interact with family members and caregivers through face-to-face interactions to facilitate learning more efficiently. Television with the purpose of educating children can allow for the learning of language, literacy, and cognitive development. Adults should be cognizant of background television in the presence of children as studies have shown that an increase in background television can adversely affect a child’s language usage, executive functioning, quality of their play, language acquisition, attention, and cognition in children younger than 5 years of age. In addition, excessively watching television may also affect mathematic skills, language and reading at a young age (Ponti et al., 2017).

Because of the increased exposure of screen time in children and the lack of research in this area, the current study aimed to gain a deeper understanding of the relationship between screen time (exposure) and the impact on language development (outcome) in children (population). Thus, this study aimed to answer the following research question: What effects does screen time have on children’s language development?

## Methodology

A scoping review was conducted to explore the available literature, on the influence of screen time on language development (Munn et al., 2018). The scoping review methodology allowed for the broad exploration of the topic and allowed the researchers to be more versatile when reporting on the diverse literature present (Colquhoun et al., 2014).

The research team followed the methods advised by Levac, Colquhoun and O’Brien (2010) and involved two researchers. These two researchers decided on the study issue, the search terms, keywords, and the databases to be searched. The researchers followed Arksey and O’Malley’s (2005) five-phase approach, which included the following steps: (1) defining the research question, (2) locating relevant publications, (3) study selection, (4) charting the data, and (5) compiling, summarising, and publishing the results. According to Peters et al. (2015), data extraction enables the researcher to create a logical and systematic summary of the literature that is related to the scoping review’s purpose, objectives, and research question. The researchers created a data extraction table to extract and retain pertinent information relating to the effects of screen usage on language development. It must be noted that this process was limited because of the university almanac and scheduling. The table contains information on the article number, the title of each research/source, author(s), year in which the source was published, the country, study’s aims and objectives, study design, participant description, sample size, and findings.

## Data sources and eligibility criteria

The initial search was carried out in July 2020 across the following electronic databases: Google Scholar, PubMed, EBSCO, JSTOR, Wiley Online Library, ScienceDirect, SAGE Journals, and SpringerLink. The eligibility criteria included studies published in English from 2000 onwards (until 2020), with an emphasis on language development and screen time. Because of a lack of translation resources, only English-language publications were included. Inclusion criteria also included full-text publications and sources.

The titles and abstracts of the articles were obtained from the above databases and a search was conducted on these databases using the following keywords and combinations: (child OR children) AND (‘screen time’ OR ‘digital devices’ OR ‘media use’ OR television OR technology) AND ‘language development’ AND ‘speech development’.

### Search strategy

The researchers analysed the titles and abstracts of the articles obtained from the databases and thereafter conducted a search on these databases using the following keywords and combinations: ‘child/children, screen time’ OR ‘digital devices’ OR ‘media use’ OR television OR technology AND; language development. The articles were required to present research on children’s exposure to screen time, television, and media, with one of the study’s objectives or components addressing the influence of screen time on language development. Finally, publications were to report on research completed globally between 2000 and 2020, to ensure that the literature was current, as concerns about screen time are new and growing as technology advances (Hawi & Rupert, 2015). Two reviewers blindly retrieved specific information from the studies to ensure their reliability.

**FIGURE 1:**
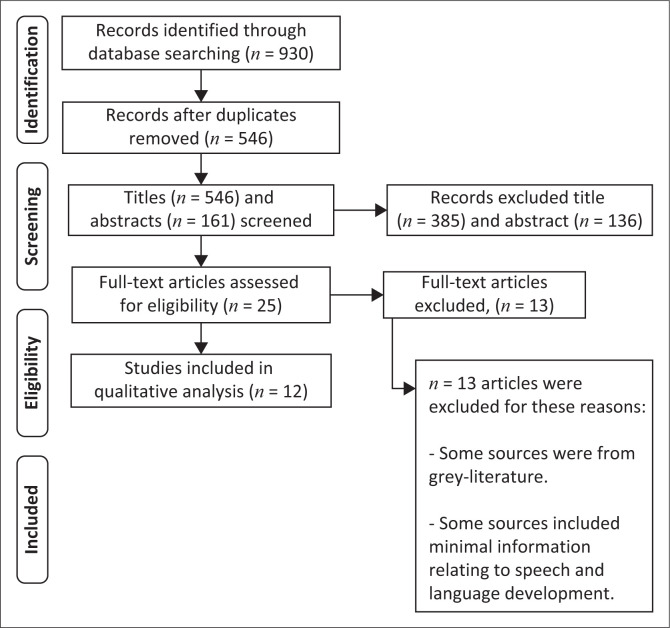
Preferred reporting items for systematic reviews and meta-analyses extension for scoping reviews flow diagram of article search results.

According to Joanna Briggs Institute (JBI) Arksey and O’Malley (2005), the first level review looked at titles, the second at abstracts, and the third at full-text articles (refer to Figure 1). Entries that did not match the study’s minimum inclusion requirements were omitted. An abstract relevance screening spreadsheet with high level reviewer agreement (overall kappa) more than 0.8 was employed by the researchers (Viera & Garrett, 2005). Source selection was done blindly to improve inter-rater consistency. Two reviewers searched relevant databases for sources and evaluated them against the inclusion/exclusion criteria. When a disagreement emerged, the third reviewer was consulted for conflict resolution to make a final decision. The reviewers screened the sources’ reference lists prior to examining the titles and abstracts for inclusion and removal.

The Internet browser (Google Chrome was recommended) was to be opened on a personal computer (PC) and the name of the database (Google Scholar, PubMed, EBSCO, JSTOR, Wiley Online Library, ScienceDirect, SAGE Journals or SpringerLink) to be entered. The ‘advanced’ search option was selected and the reviewer typed the following key terms: (child OR children) AND (‘screen time’ OR ‘digital devices’ OR ‘media use’ OR television OR technology) AND ‘language development’ AND ‘speech development’. Thereafter under filters, the years between 2000 and 2020 were selected under the publication date, with English and ‘peer-reviewed’ being selected before clicking search. The screening protocols were then utilised when going through the search results.

### Data charting and analysis

Thematic analysis was used since it is the most powerful and effective method of qualitative data analysis because it captures the complex meanings contained within texts (Guest et al. 2011). Clarke and Braun’s (2013) six-phase theme analysis plan was employed in this investigation.

### Ethical considerations

This article followed all ethical standards for research without direct contact with human or animal subjects.

## Results

### Search results

Figure 1 depicts the PRISMA-ScR flow diagram showing the data collected by searching through the various databases. A total of 930 articles were identified in the initial searches. After removing the duplicate studies, the total number of studies to be screened was 546. Following the title and abstract screening, 385 and 136 articles were excluded, respectively, as they did not meet the inclusion criteria and the screening criteria. Therefore, 25 full-text articles were screened with 13 of these articles being excluded as certain articles were grey literature, which falls part of the exclusion criteria, and because of the articles containing minimal information relating to language development. The remaining 12 articles included in the review for analysis are summarised in the data extraction table (Figure 1) and Table 1. The researchers analysed the data gathered from the literature and identified themes. The reviewers utilised Mendeley Data for data management and to arrange the various sources obtained.

**TABLE 1:** Summary of the location and study designs of the included studies.

Title of study	Authors and year of publication	Country	Study design
A preliminary study on the relationship between characteristics of television content and delayed speech development in young children	Kanako Okuma and Masako Tanimura (2009)	Tokyo, Japan (Asia)	Observational study
Association of screen time use and language development in Hispanic toddlers: A cross-sectional and longitudinal study	Helena Duch, Elisa M. Fisher, Ipek Ensari, Marta Font, Alison Harrington, Caroline Taromino, Jonathan Yip and Carmen Rodriguez (2013)	-	Cross-sectional and longitudinal study
Association between media viewing and language development in children under age 2 years	Frederick J. Zimmerman, Dimitri A. Christakis and Andrew N. Meltzoff (2007)	United States of America (North America)	Survey-based
Do hours spent viewing television at ages 3 and 4 predict vocabulary and execution functioning at age 5?	A. Nayena Blankson, Marion O’Brien, Esther M. Leerkes, Susan D. Calkins and Stuart Marcovitch (2015)	-	Longitudinal study
Duration of watching television and child language development in young children	Silva Audya Perdana, Bernie Endyami Medise and Emi Hemawati Purwaningsih (2017)	Jakarta, Indonesia (Asia)	Cross-sectional study
How does the use of modern communication technology influence language and literacy development? A review	Helen J. Watt (2010)	United Kingdom (Europe)	Review
Infants’ and toddlers’ television and language outcomes	Deborah L. Linebarger and Dale Walker (2005)	United States of America (North America)	A longitudinal process-product design
Relationship between television viewing and language delay in toddlers: Evidence from a Korea national cross-sectional survey	Haewon Byeon and Saemi Hong (2015)	Korea (Asia)	Cross-sectional survery
Screen media and language development in infants and toddlers: An ecological perspective	Deborah L. Linebarger, Sarah E. Vaala (2010)	United States of America (North America)	Developmental review
Television and very young children	Daniel R. Anderson and Tiffany A. Pempek (2005)	United States of America (North America)	Review
Television viewing associates with delayed language development	Weerasak Chonchaiya and Chandhita Pruksananonda (2008)	Thailand (Asia)	Case-control study
Television viewing in Thai infants and toddlers: impacts to language development and parental perceptions	Nichara Ruangdaraganon, Jariya Chuthapisith, Ladda Mo-suwan, Suntree Kriweradechachai, Umapom Udomsubpayakul and Chanpen Choprapawon (2009)	Thailand (Asia)	Longitudinal birth cohort study (birth–2 years old)

### Characteristics of the included sources

Majority of the sources aimed at evaluating the relationship between language and screen time. Two sources examined the language used by children when communicating through technology and evaluated television and young children in relation to the American Academy of Paediatrics (AAP) guidelines that state that children below 2 years of age should not be exposed to screen time. These two sources differed from the other sources, that is, screen time was not accounted for.

A summary of the study locations and study designs utilised in the included studies is provided in Table 1. Majority of the sources reported on television as the type of screen media; one source reported on videos and another source reported on television and videos. It can be inferred that screen time not only involves television but also computers, smart phones, videos, DVDs, etc.

## Discussion

Recently a rise in screen time has occurred. Parents use screen time when concerned about their infant’s development, when parental interaction is reduced (Blankson et al., 2015; Byeon & Hong, 2015; Perdana, Medise, & Purwaningsih, 2017), and to provide opportunities for learning and to improve cognition (Hinkley & McCann, 2018).

**FIGURE 2:**
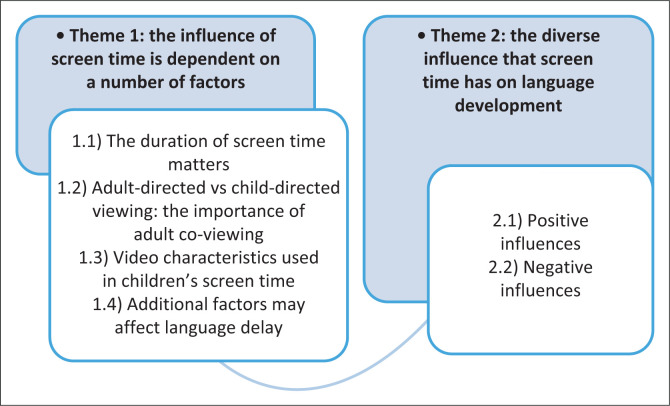
Diagram of themes and sub-themes identified.

In the current scoping review, two themes and six sub-themes were generated (see Figure 2).

Many factors exist that may affect the link between screen time and language development, which include the quantity of home communication provided, genetics (Zengin-Akkus, Celen-Yoldas, Kurtipek, & Ozmert, 2018), parent-child interaction (Blankson, O’Brien, Leerkes, Calkins, & Marcovitch, 2015; Byeon & Hong, 2015; Linebarger & Vaala, 2010), and male gender (Okuma & Tanimura, 2009).

Duch et al. (2013) reported that children who have a television in their room spend more time watching television compared to their counterparts. This is important as the amount of time spent exposed to screen time may influence language development. The study by Perdana et al. (2017) concurred with the study by Duch et al. (2013) and Zengin-Akkus et al. (2018) which stated that no relationship exists between a child’s sex, the existence of a television in children’s bedrooms, and delayed language development. Furthermore, child characteristics (existing vocabulary, age, etc.) and environmental contexts (interacting with the same content repeatedly and for a long period of time, adult co-viewing and the quantity and quality of adult–child interactions) can also influence language (Linebarger & Vaala, 2010).

Videos may refer to YouTube videos, videos on various screens, etc. Several studies show that stimulus characteristics can also influence language development. It is advisable to consider video characteristics when deciding on a programme for children. Videos that are rapidly paced with fewer close-ups, flashing/changing images, reduced language, and increased frame rate can cause language delays. Rapidly paced videos may be cognitively burdening for children (Duch et al., 2013; Linebarger & Vaala, 2010). Linebarger and Vaala (2010) stated that programmes like *Blue’s Clues* and *Dora the Explorer* have a positive effect on vocabulary, while *Teletubbies* have a negative effect on expressive language because of the video characteristics present.

Finance and neighbourhood safety influence the duration of screen time which children are exposed to. A study conducted in Soweto, South Africa, and the US reported that children viewed a larger amount of television if the neighbourhood was perceived to be more dangerous as opposed to a safer neighbourhood, as children were safer indoors compared to playing outdoors where it could be unsafe (Balton et al., 2019).

According to Perdana et al. (2017), as children grow older, the duration of screen time increases. This implies that a direct relationship exists between the duration of screen time and age. The duration of screen time is also dependent on cultural and socio-economic factors, and may be affected and influenced by the habits of family members and parents of the child. Several articles report a link between the duration of screen time and receptive and expressive language delays (Byeon & Hong, 2015; Duch et al., 2013, Perdana et al., 2017). This shows that increased exposure to screen time in children is not recommended.

According to Hudon, Fennell and Hoftyzer (2013), the quality of the programme may affect language development more than the duration of time spent watching the programme. Background television refers to content, which may not be understandable, resulting in poorer cognition as little to no attention is paid to it, and parent-child interaction is reduced which is important for cognitive development (Anderson & Subrahmanyam, 2017; Pempek, Kirkorian, & Anderson, 2014). As previously mentioned, excessive background television may have adverse effects on children. It is distractive, disrupts playtime and results in slower language development and lower vocabulary scores as parent-child interaction is reduced.

Screen time may occur when supervised by an adult (adult-directed) or by the child independently (child-directed). Adults are expected to ensure that their children have access to linguistically and age-appropriate content (Watt, 2010). Watching two or more hours of child-directed TV a day showed 6.25 times greater vulnerability to lower communication scores as compared to the same amount of adult-directed TV (Duch et al., 2013). This implies that child-directed viewing may negatively impact a child making adult-directed viewing the preferred option. Co-viewing encourages language acquisition. Screen time can help promote learning, but not as much as what can be learnt through social interactions (Strouse, Troseth, O’Doherty, & Saylor, 2018). The value of a competent adult, such as parents, caregivers, or siblings, providing stimulation by means of posing questions and interacting with the child during screen media is highlighted as this will promote vocabulary, vocalisations, and comprehension. Often televisions and screen media are used as ‘babysitters’ to distract children when parents are busy or when they are not present (Nikken, 2019). This implies that a competent adult may not always be present and that viewing may be child-directed. This may influence children’s language development.

According to Kemp (2021), 98.2% of people in South Africa have a mobile phone, 98% have a smart phone, 85.4% have a laptop/computer, 43.2% have a tablet, 16.7% have a streaming television or device, and 18.8% have a smart watch. These statistics are important as it assists in understanding the percentage of individuals who own the above devices.

Language develops early through interaction with parents and caregivers who provide the child with the means to learn the forms and features of the language (Blankson et al., 2015; Byeon & Hong, 2015). In South Africa, many people use English as a second language. Research on the impact of media which differs from the child’s first languages indicates that children may be negatively impacted and places the child 14.7 times more at risk of a language delay. This is because of the difference in language and grammatical order of the language which may be confusing for the child and reduces the amount of guidance provided by the parents during co-viewing (Perdana et al., 2017).

The age of exposure to screen time may result in language delay. According to the APA, children below the age of two should not be watching television (Perdana et al., 2017). This is because children should comprehend the concept of dual representation which begins to develop around the age of two and is not fully developed until after the child is 2 years old (Linebarger & Vaala, 2010).

Screen time has positive effects on a child’s cognitive abilities, higher-order language, and literacy skills (Blankson et al., 2015; Watt, 2010). Screen time may increase vocabulary and language production skills in two-and-a-half-year-old children (Linebarger & Vaala, 2010). Several experiments show that sufficient repetitive exposure to a programme improves a child’s problem-solving abilities, ability to imitate and the ability to learn new words (Linebarger & Vaala, 2010). Repeated exposure allows for processing difficulties to be overcome and facilitates learning of vocabulary and content (Linebarger & Vaala, 2010). However, children below 22 months old were not able to learn novel words with repeated exposure to a child-directed television programme, but were able to learn similar new words within their natural environment (Krcmar, 2014). Increased exposure to stimuli that is absorbed but not developmentally constructive may influence brain development and language acquisition (Watt, 2010). ‘Heavy’ use may negatively impact attention that may affect the child’s literacy with these adverse effects outweighing the advantages to cognition and literacy (Watt, 2010).

Screen time can negatively affect language acquisition, early language development (Byeon & Hong, 2015) and play (Lin, Cherng, Chen, Chen, & Yang, 2015). The first 3 years of a child’s development are important for brain development, which is affected by environmental influences (Watt, 2010). Literacy is also affected as spell-check gadgets reduce spelling abilities with technological communication facilitating spelling errors to go unnoticed (Watt, 2010). It is the role of Speech-language therapists (SLTs) to identify, assess and manage individuals presenting with speech and language difficulties (Kathard et al., 2011).

## Potential study implications

This study may contribute to limited research as the impact of screen time on children’s language development has not been sufficiently researched, and the sources included in the analysis were not within the South African context. Current guidelines relating to screen time are set by the World Health Organization (WHO) and APA, and correlate with that from Canada, Australia, and South Africa. It states that children below 2 years of age should not be exposed to screen time, children between the ages of 2 and 4 years should not exceed one hour daily, and 2 h per day should not be exceeded in 5- to 17-year-olds. This scoping review may contribute to guidelines relating to language development of children exposed to screen time and will inform future research exploring the relationship between screen time and language development. Future studies could include specific socio-economic factors and a range of languages and cultures. An assessment could also be conducted with children to assess the impact of screen time on their language development considering parents’ perceptions.

This scoping review may have clinical implications and has the potential to guide speech-language therapy. It could also provide insight relating to guidance and recommendations for parents. Speech Language Therapists should provide parents with strategies and skills to promote language stimulation and the importance of co-viewing (Canadian 24–Hour Movement Guidelines, 2020; The Conversation, 2019; WHO, 2019).

As schools move away from paper-based activities (Shonfeld & Meishar-Tal, 2017), this study could inform education policies with regards to the increased duration of time spent with screen time which may have adverse effects on language development.

## Conclusion

This review has provided valuable information on the influence of screen time on language development. The review revealed that the influence of screen time is multifactorial and includes both positive and negative influences on children’s language development. Majority of the studies analysed indicate that an increase in the amount of screen time and the early age of onset of viewing has negative effects on language development, especially for the children under the age of two with older age of onset of viewing showing some benefits. In addition, video characteristics, content and co-viewing also influence language development. Parents play a critical role in language development. Although, there are both positive and negative effects. It appears that the negative influences outweigh the positive influences, and the researchers invite further enquiry into this area.
